# Understanding empathy deficits and emotion dysregulation in psychopathy: The mediating role of alexithymia

**DOI:** 10.1371/journal.pone.0301085

**Published:** 2024-05-08

**Authors:** Matthias Burghart, Alexander H. J. Sahm, Sergej Schmidt, Jan Bulla, Daniela Mier

**Affiliations:** 1 Department of Psychology and Psychotherapy, University of Konstanz, Konstanz, Germany; 2 Forensic Psychiatry and Psychotherapy, Reichenau Psychiatric Center, Reichenau, Germany; 3 Department of Forensic Psychiatry and Psychotherapy, University of Ulm, Ulm, Germany; University of Padova, ITALY

## Abstract

Psychopathy is a severe personality disorder marked by a wide range of emotional deficits, including a lack of empathy, emotion dysregulation, and alexithymia. Previous research has largely examined these emotional impairments in isolation, ignoring their influence on each other. Thus, we examined the concurrent interrelationship between emotional impairments in psychopathy, with a particular focus on the mediating role of alexithymia. Using path analyses with cross-sectional data from a community sample (*N* = 315) and a forensic sample (*N* = 50), our results yielded a statistically significant mediating effect of alexithymia on the relationship between psychopathy and empathy (community and forensic) and between psychopathy and emotion dysregulation (community). Moreover, replacing psychopathy with its three dimensions (i.e., meanness, disinhibition, and boldness) in the community sample revealed that boldness may function as an adaptive trait, with lower levels of alexithymia counteracting deficits in empathy and emotion dysregulation. Overall, our findings indicate that psychopathic individuals’ limited understanding of their own emotions contributes to their lack of empathy and emotion dysregulation. This underscores the potential benefits of improving emotional awareness in the treatment of individuals with psychopathy.

## Introduction

Healthy emotional functioning is essential for human behavior. The ability to recognize, understand, and manage one’s own emotions, as well as the ability to recognize, empathize with, and respond appropriately to the emotions of others, allows one to build and maintain relationships, cope with stress and challenges, and make sound decisions [1]. Impairments in these skills are key symptoms of psychopathy, including a lack of empathy (e.g., [2,3]), alexithymia (e.g., [2,4,5]), and emotion dysregulation (e.g., [6–8]). However, previous studies have generally examined these deficits in isolation and ignored their influence on each other [9,10], thus failing to account for the complex interrelationship between emotional processes [11,12]. The aim of the present study is to uncover the interplay between emotional deficits in psychopathy and the mediating role of alexithymia, using a forensic and a community sample.

## Psychopathy

Psychopathy as defined by Robert Hare is regarded as one the most important concepts in forensic psychology [13,14]. It is marked by a lack of remorse, shallow emotions, and a tendency to engage in manipulative and risky behavior [15]. Psychopathic individuals are often perceived as charismatic and charming, but in reality, tend to be manipulative, deceitful, and prone to antisocial behavior [15,16]. Although the etiology of psychopathy is not yet fully understood, it is likely the result of a complex interaction between genetic [17] and environmental [18,19] risk factors.

Psychopathy is diagnosed with the Psychopathy Checklist-Revised (PCL-R; [20]), a semi-structured interview. The PCL-R divides psychopathy into two factors (i.e., Factor 1: Interpersonal/Affective and Factor 2: Social Deviance) and four facets (i.e., Interpersonal, Affective, Lifestyle, and Antisocial). Because of its historical impact and thorough assessment process, it is widely recognized as the gold standard for assessing psychopathy [21]. However, in recent decades, additional self-report instruments have emerged that not only offer less time-intensive assessments but also place less emphasis on antisocial behavior as a defining characteristic of psychopathy, and are thus suitable for use with community samples. One such instrument is the Triarchic Psychopathy Measure (TriPM; [22]), which is based on the Triarchic Model of Psychopathy [23]. According to this model, psychopathy is a multidimensional construct that encompasses three distinct dimensions: Disinhibition (i.e., tendencies towards impulsivity, impaired affect regulation, poor planning ability, hostility, and mistrust), meanness (i.e., deficient empathy and a lack of close attachments combined with exploitative behavior and cruelty towards others), and boldness (i.e., capacity to remain calm under pressure, emotional resilience, confidence, and high social assertiveness, [23]). A growing number of studies support the validity and reliability of the TriPM and its factor structure in both incarcerated and community samples [24–26].

## Empathy, alexithymia, and emotion dysregulation in psychopathy

In the following section, we introduce the constructs empathy, alexithymia, and emotion regulation and review the literature linking them to psychopathy.

### Empathy

The term empathy is often used intuitively in everyday language to describe the sharing of feelings, but definitions within the scientific community remain heterogenous [27]. Nevertheless, there is consensus on its multifaceted structure, which encompasses two conceptually distinct domains, namely affective and cognitive empathy [27,28]. The former describes the ability to emotionally share and experience another person’s feelings, while the latter refers to the ability to correctly identify and understand the emotional states or feelings of others [29].

Despite a general lack of empathy being considered a defining trait of psychopathy [30], there is ongoing debate about the extent to which each of the two domains of empathy are impaired. A commonly proposed theory is that psychopathic individuals lack affective empathy, but exhibit normal cognitive empathy [31–35]. This could explain their inability to empathize with others emotionally, yet still being able to charm and manipulate them, as this requires the understanding of others’ thoughts and feelings. However, this view has been challenged by two recent meta-analyses that revealed significant deficits in both domains, albeit with slightly larger effect sizes for affective empathy [2,36].

### Alexithymia

The literal meaning of alexithymia is “no words for emotions”. It is characterized by the inability to identify and describe feelings, as well as by an externally-oriented thinking style (i.e., the tendency to focus on external stimuli and events rather than internal experiences; [37]). First introduced in the 1970s, alexithymia has now been linked to a variety of mental and physical health problems, including depression [38], anxiety [39], and somatic complaints [40].

Alexithymia has received considerable attention in psychopathy research in recent years. Studies have yielded positive correlations between psychopathy and all three aspects of alexithymic symptoms (i.e., difficulty identifying and describing feelings, and externally-oriented thinking; [2]). However, these relationships are stronger for factor 2 than factor 1 of psychopathy, and stronger in women than in men [2].

### Emotion regulation

In its broadest sense, emotion regulation (ER) refers to “shaping which emotions one has, when one has them, and how one experiences or expresses these emotions” [41]. This includes all strategies aimed at altering emotional states [41]. In recent decades, various strategies have been identified, with cognitive reappraisal and expressive suppression being the most studied [42]. The former involves the cognitive reinterpretation of an emotional event in order to change the emotional response it generates (e.g., telling yourself that the rude cashier probably just had a bad day; [43,44]). Suppression, on the other hand, refers to inhibiting the outward expression of an already fully generated emotion (e.g., showing a poker face despite being nervous; [43–45]). Although both strategies can effectively change emotional states in the short term, research suggests that the general use of reappraisal is more adaptive than suppression, as it leads to more desirable emotions without long-term costs [46]. In contrast, habitual use of suppression has been associated with violent behavior [47,48], weaker social relationships [1,49], feelings of inauthenticity [45], and lower emotional well-being [45,50,51].

Although research on the relationship between ER and psychopathy is scarce, the available studies suggest an association between psychopathy and emotion dysregulation, as demonstrated in both offender and community samples [52–55]. In addition, individuals with higher levels of psychopathic traits are more likely to use suppression over reappraisal compared to individuals with lower levels of these traits [56].

## The mediating role of alexithymia

As emphasized in the outset of this article, meta-analytical reviews have linked psychopathy to empathy deficits [2,36], alexithymia [2], and emotion dysregulation [56]. However, these associations have generally been investigated in isolation from each other, despite several theoretical models postulating a mediating role of alexithymia in the interaction of these emotional concepts.

For instance, the Self to Other Model of Empathy [11] and the Introspection-Centric Simulation Theory [12] both suggest that the ability to consciously represent and interpret one’s own emotions (i.e., the absence of alexithymia) is essential for experiencing empathy. This is because introspection of an isomorphic internal state provides essential cues for the correct interpretation of other’s internal states [11,12]. Therefore, the less a person understands their own emotions, the less they will be able to empathize with others. This view is supported by a plethora of studies indicating a negative relationship between alexithymia and affective as well as cognitive empathy [32,57–64].

Similar to how the ability to understand one’s own emotions is a prerequisite for experiencing empathy, it is also considered a prerequisite for effective ER [65–68]. If a person is feeling angry, for example, but cannot identify that emotion, they may struggle to effectively regulate their current emotional state. Indeed, previous research has uniformly found a positive relationship between alexithymia and emotion dysregulation, corroborating this notion [5,68–73]. In addition, alexithymic individuals have been shown to resort to suppression more often than to reappraisal during ER [74–77], which is expected considering that reappraisal requires explicit engagement with one’s feelings, while suppression does not [45].

### The present study

Drawing on these findings, we explored the interrelationship of emotional deficits in psychopathy with a particular focus on whether alexithymia mediates the association between psychopathy and empathy deficits as well as between psychopathy and emotion dysregulation. To maximize the variation of psychopathic traits and emotional impairments, we conducted our study in a community and a forensic sample. This not only improves the generalizability of our findings, but also allows us to determine whether the results extend to both community-dwelling individuals and psychiatric patients with a history of criminal behavior.

## Method

### Procedure and participants

Data was collected in 2022 in two independent samples, including individuals from the community and from a forensic hospital. The present study was approved by the ethics committee of the University of Konstanz (protocol number: 33/2021, “Einflussfaktoren auf Empathie”), and all participants gave their written informed consent prior to participation.

### Community sample

Participants were recruited via SONA (i.e., study distribution service of the University of Konstanz), flyers distributed in local stores, and social media. Interested participants were given access to the Qualtrics online survey. Upon completion, participants had the option to participate in a raffle (3 * 50 €) or to receive course credits. To ensure data quality, individuals who did not complete the survey (*n* = 72), failed the attention check (*n* = 2), or were younger than 18 years (*n* = 2) were removed. No additional exclusion criteria were applied.

The final sample consisted of 315 participants (68% female, 1% diverse). Their ages ranged from 18 to 62 years (*M* = 24.19, *SD* = 7.47). The educational level of all participants was distributed as follows: Lower secondary education (< 1%), secondary education or vocational training (4%), upper secondary education (67%), bachelor or equivalent (20%), and master or equivalent (7%). Twenty-three percent of the sample reported to have suffered from a mental illness in the past.

### Forensic sample

Forensic patients were recruited from four different wards at the Reichenau Centre of Psychiatry (*Zentrum für Psychiatrie Reichenau)*. According to the German Criminal Code (*StGB*), these patients were placed in a forensic hospital either because they had committed an offense in a state of incapacity or reduced culpability and still posed a danger to society (§ 63 StGB; *n* = 31) or because they had committed an offense under the influence of alcohol or drugs or as a result of a substance addiction (§ 64 StGB; *n* = 19). The data was collected by SM who visited all wards and presented the study details. Before participation, the eligibility of each interested patient was discussed with the clinical staff to confirm the absence of acute psychiatric symptoms and intellectual disabilities, an acceptable level of German language proficiency, and the ability to give written informed consent. Inpatients were approved to take part in the study only if they met these criteria. Questionnaires were administered on each ward in a separate, quiet room with up to two participants at a time. The completion of all questionnaires took an average of 30 minutes and was compensated with 10€.

The sample included 50 forensic inpatients (16% female; no participants had to be excluded) between the ages of 20 to 60 years (*M* = 34.90, *SD* = 9.98). Participants reported the following levels of education: No completed education (8%), lower secondary education (30%), secondary education or vocational training (44%), and upper secondary education (18%). Due to the need to ensure complete anonymization during data collection and the small sample size compared to the complexity of the path models tested, no information on mental disorders or criminal offenses was assessed. That said, all inpatients meet the criteria for a mental disorder, as this is a requirement for admission to a forensic hospital under the German Criminal Code. A recent study across forensic hospitals in the German federal state of Baden-Württemberg found that schizophrenia spectrum disorders, personality disorders, and substance abuse disorders are among the most common diagnoses [78]. Consequently, treatment plays a crucial role in these facilities, with the Reichenau Centre of Psychiatry implementing psychotherapy through both individual and group sessions several times a week, which is further complemented by pharmacotherapeutic interventions.

## Measures

Both samples received the same four self-report measures, with the difference that the community sample completed them online, while the forensic inpatients received the paper-pencil versions. All questionnaires were administered in their official German translation. The Cronbach’s alpha coefficients attained in the present study are reported in Table 1.

**Table 1:** Cronbach’s alpha coefficients for all included measures.

Scale	Cronbach’s *α*	
	Community Sample	Forensic Sample
TriPM	.84	.88
Meanness	.84	.87
Disinhibition	.82	.86
Boldness	.82	.72
SPF-IRI	.76	.67
Empathic Concern	.72	.49
Perspective Taking	.72	.74
Personal Distress	.74	.64
Fantasy	.77	.59
TAS-20	.87	.82
Difficulty Describing Feelings	.83	.65
Difficulty Identifying Feelings	.87	.86
Externally-Oriented Thinking	.68	.49
ERQ		
Reappraisal	.83	.83
Suppression	.73	.56

TriPM = Triarchic Psychopathy Measure; SPF-IRI = Saarbrücker Persönlichkeitsfragebogen; TAS-20 = Toronto Alexithymia Scale-20; ERQ = Emotion Regulation Questionnaire. For the ERQ, Cronbach’s *α* is only reported for the two subscales, as no total score is computed.

### Psychopathy

Psychopathic traits were assessed with the TriPM [22] (German translation: [79]). This self-report measure comprises 58 items that are rated on a 4-point Likert scale. Item scores are combined to three subscales: Meanness, boldness, and disinhibition. Higher scores on a subscale indicate greater expression of the respective psychopathic traits. A psychopathy composite score was calculated by adding up the subscales. Cronbach’s alpha coefficients ranged from acceptable (*α* > .70) to good (*α* > .80) in both samples.

### Empathy

Empathy was measured with the Saarbrücker Persönlichkeitsfragebogen (SPF-IRI; [80]), which is a shortened and reworked version of the widely used Interpersonal Reactivity Index (IRI; [81]). It measures the same four empathy facets, namely: Empathic concern (EC), perspective taking (PT), personal distress (PD), and fantasy (FS). The SPF-IRI contains 16 items that are rated on a 5-point Likert scale. EC captures other-oriented feelings such as compassion or concern for a suffering individual. PT measures the ability to take over the perspective of another person. PD refers to self-oriented aversive feelings in response to the suffering of others. FS assesses the tendency to put oneself into the role of fictional characters in books and movies [81]. Although the IRI is not intended to provide a composite empathy score, the SPF-IRI has been shown to adequately capture overall empathy by summing PT, EC, and FS [80]. All Cronbach’s alpha coefficients were acceptable (*α* > .70) in the community sample, but poor (*α* > .50) to questionable (*α* > .60) in the forensic sample, with EC being unacceptable (*α* = .49). The total score performed better than most of the individual subscales of the IRI in the forensic sample (*α* = .67).

### Alexithymia

The Toronto Alexithymia Scale-20 (TAS-20; [37]; German translation: [82]) was used to assess alexithymia. The TAS-20 is a 20-item self-report measure that captures the three symptom domains of alexithymia: Difficulty describing feelings (DDF), difficulty identifying feelings (DIF), and externally-oriented thinking (EOT). Each item is rated on a 5-point Likert scale with higher scores indicating the presence of more alexithymic symptoms. The total score was obtained by adding up all subscales. The internal consistency of the TAS-20 total score and its subscales was generally good (*α* > .80) in both samples. However, the performance of EOT was questionable (*α* = .68) and unacceptable (*α* > .49) among participants from the community and the forensic hospital, respectively.

### Emotion regulation

The habitual use of cognitive reappraisal and expressive suppression was examined with the Emotion Regulation Questionnaire (ERQ; [45]; German translation: [83]), which includes 10 items rated on a 7-point Likert scale. Larger scores on either scale (i.e., reappraisal and suppression) suggest a tendency toward the respective ER strategy. In both samples, reappraisal yielded good (*α* > .80) Cronbach’s alpha coefficients. In contrast, the coefficients for suppression were acceptable (*α* = .73) in the community sample, but poor (*α* = .56) in the forensic sample.

## Data analysis

Differences between the two samples on all measured variables were assessed using ANCOVAs, with age, sex, and education (in years) as covariates. In addition, their bivariate associations within each sample were examined with zero-order correlation coefficients.

Path analyses were conducted to determine whether the relationship between psychopathy and empathy and between psychopathy and emotion dysregulation is mediated by alexithymia. Two separate models were tested for each sample, one with only the TriPM total score as a predictor and the other with the three TriPM factors. Standard guidelines were followed to compute mediation effects, with the Sobel method and the maximum likelihood estimator used for all analyses [84]. As recommended by Hayes [85], confidence intervals were estimated via non-parametric bootstrapping with 5,000 iterations. To interpret the magnitude of a mediation effect, the completely standardized indirect effect (ab_cs_) was compared to the following benchmarks: .01 = small effect, .09 = medium effect, and .25 = large effect [86]. Since our survey used a forced-choice design, there was no missing data. All analyses were performed with the latest versions of R [87] and JASP [88] using the *lavaan* package [89].

## Results

The forensic sample differed from the community sample in age and education, with forensic inpatients being on average older (*t*(363) = 8.95, *p* < .001) and less educated (in years; *t*(363) = –‍11.38, *p* < .001) than participants from the community. Additionally, there was a significant difference in gender distribution between the two groups (*X*^2^(1) = 49.27, *p* < .001), insofar as the community sample comprised more female participants than the forensic sample (note: individuals who identified as “diverse” in the community sample were excluded in the Chi-squared test, as no participants indicated “diverse” in the forensic sample).

Forensic inpatients reported statistically significantly more psychopathic traits (total: *η*^2^ = .115, *p* < .001; meanness: *η*^2^ = .031, *p* < .001; disinhibition: *η*^2^ = .145, *p* < .001; boldness: *η*^2^ = .015, *p* = .016). No significant differences were found in the level of reported empathy, alexithymia, or habitual use of ER strategies (Table 2).

**Table 2:** Means, standard deviations (SD), and group comparisons (controlled for age, sex, and education) for all study variables.

	Mean (SD)		*F*	*η* ^2^
	Community Sample (*N* = 315)	Forensic Sample (*N* = 50)		
TriPM total	109.78 (14.47)	136.16 (20.85)	50.86***	.115
Meanness	29.98 (7.24)	38.34 (9.75)	12.39***	.031
Disinhibition	33.52 (7.12)	48.16 (11.40)	63.22***	.145
Boldness	46.29 (8.17)	49.66 (7.61)	5.81*	.015
SPF-IRI total	43.49 (6.82)	40.50 (6.77)	.23	.001
EC	14.87 (2.88)	14.40 (2.67)	.44	.001
PT	14.37 (2.78)	13.76 (3.41)	1.37	.004
PD	11.57 (3.26)	10.70 (3.21)	.05	.000
FS	14.24 (3.28)	12.34 (3.21)	.28	.001
TAS-20 Total	46.41 (11.99)	47.16 (11.21)	.41	.001
DDF	13.04 (4.58)	12.78 (4.06)	2.98	.008
DIF	16.72 (5.94)	14.70 (5.89)	.92	.002
EOT	16.65 (4.54)	19.68 (4.41)	1.69	.004
ERQ Reappraisal	4.54 (1.12)	4.38 (1.39)	.14	.000
ERQ Suppression	3.66 (1.29)	3.92 (1.26)	.15	.000

TriPM = Triarchic Psychopathy Measure; SPF-IRI = Saarbrücker Persönlichkeitsfragebogen; EC = empathic concern; PT = perspective taking; PD = personal distress; FS = fantasy; TAS-20 = Toronto Alexithymia Scale-20; DDF = difficulty describing feelings; DIF = difficulty identifying feelings; EOT = externally-oriented thinking; ERQ = Emotion Regulation Questionnaire; *F* = statistics based on an ANCOVA with age, sex, and education (in years) as covariates; *η*^2^ = effect size.

*** *p* < .001

** *p* < .01

* *p* < .05.

Zero-order correlation coefficients for the community sample and the forensic sample are shown in Tables 3 and 4, respectively. While empathy exhibited a negative relationship with suppression (*r*_community_ = –.15, *p* < .01) and a positive relationship with reappraisal (*r*_community_ = .31, *p* < .001), the reverse was found for alexithymia (suppression: *r*_community_ = .49, *p* < .001; reappraisal: *r*_community_ = –.32, *p* < .001). Empathy and alexithymia were negatively associated (*r*_community_ = –.30, *p* < .001). Overall, psychopathy was negatively related to empathy (*r*_community_ = –.29, *p* < .001) and reappraisal (*r*_community_ = –.14, *p* < .05) as well as positively related to alexithymia (*r*_community_ = .31, *p* < .001) and suppression (*r*_community_ = .13, *p* < .05). However, when considering psychopathy factors, the directions of these associations differed, with boldness showing a positive correlation with cognitive empathy (i.e., perspective taking; *r*_community_ = .16, *p* < .01) and reappraisal (*r*_community_ = .14, *p* < .05), and a negative correlation with alexithymia (*r*_community_ = –.23, *p* < .001) and suppression (*r*_community_ = –.12, *p* < .05).

**Table 3:** Zero-order correlations across all study variables for the community sample (*N* = 315).

	1.	2.	3.	4.	5.	6.	7.	8.	9.	10.	11.	12.	13.	14.	15.
1. TriPM	–														
2. MEAN	.79***	–													
3. DIS	.62***	.46***	–												
4. BOLD	.53***	.11	–.18**	–											
5. SPF-IRI	–.29***	–.51***	–.16**	.06	–										
6. EC	–.34***	–.55***	–.10	–.01	.80***	–									
7. PT	–.17**	–.37***	–.15**	.16**	.69***	.36***	–								
8. PD	–.12*	.03	.39***	–.58***	.01	.08	–.09	–							
9. FS	–.18**	–.25***	–.11	.01	.79***	.49***	.26***	.02	–						
10. TAS-20	.31***	.40***	.49***	–.23***	–.30***	–.23***	–.26***	.45***	–.21***	–					
11. DDF	.18**	.28***	.30***	–.18**	–.25***	–.26***	–.11	.31***	–.20***	.85***	–				
12. DIF	.20***	.23***	.53***	–.31***	–.10	–.03	–.14*	.55***	–.06	.84***	.61***	–			
13. EOT	.38***	.47***	.30***	–.01	–.42***	–.31***	–.39***	.15**	–.28***	.69***	.43***	.30***	–		
14. REAP	–.14*	–.27***	–.17**	.14*	.31***	.21***	.28***	–.13*	.21***	–.32***	–.29***	–.22***	–.28***	–	
15. SUPP	.13*	.26***	.15**	–.12*	–.15**	–.24***	.00	.15**	–.11	.49***	.60***	.28***	.32***	–.12*	–

*N* = 315; TriPM = Triarchic Psychopathy Measure; MEAN = meanness; DIS = disinhibition; BOLD = boldness; SPF-IRI = Saarbrücker Persönlichkeitsfragebogen; EC = empathic concern; PT = perspective taking; PD = personal distress; FS = fantasy; TAS-20 = Toronto Alexithymia Scale-20; DDF = difficulty describing feelings; DIF = difficulty identifying feelings; EOT = externally-oriented thinking; REAP = cognitive reappraisal; SUPP = expressive suppression

*** *p* < .001

** *p* < .01

* *p* < .05.

**Table 4:** Zero-order correlations across all study variables for the forensic sample (*N* = 50).

	1.	2.	3.	4.	5.	6.	7.	8.	9.	10.	11.	12.	13.	14.	15.
1. TriPM	–														
2. MEAN	.89***	–													
3. DIS	.80***	.63***	–												
4. BOLD	.41**	.23	–.12	–											
5. SPF-IRI	–.35*	–.39**	–.43**	.19	–										
6. EC	–.40**	–.42**	–.34*	–.02	.62***	–									
7. PT	–.25	–.32*	–.34*	.25	.81***	.30*	–								
8. PD	–.18	–.20	.26	–.64***	–.01	.08	–.09	–							
9. FS	–.14	–.13	–.25	.16	.74***	.16	.40**	.02	–						
10. TAS-20	.37**	.40**	.51***	–.27	–.49***	–.43**	–.45**	.23	–.21	–					
11. DDF	.14	.21	.26	–.26	–.49***	–.33*	–.46***	.21	–.26	.86***	–				
12. DIF	.39**	.34*	.49***	–.09	–.30*	–.22	–.33*	.18	–.09	.79***	.52***	–			
13. EOT	.28*	.38**	.41**	–.33*	–.41**	–.49***	–.28	.17	–.17	.70***	.55***	.20	–		
14. REAP	.08	.00	.06	.13	.02	–.01	.11	–.21	–.06	–.04	–.03	–.01	–.05	–	
15. SUPP	.08	.04	.21	–.15	–.17	–.15	–.09	.06	–.13	.20	.21	.08	.19	.11	–

*N* = 50; TriPM = Triarchic Psychopathy Measure; MEAN = meanness; DIS = disinhibition; BOLD = boldness; SPF-IRI = Saarbrücker Persönlichkeitsfragebogen; EC = empathic concern; PT = perspective taking; PD = personal distress; FS = fantasy; TAS-20 = Toronto Alexithymia Scale-20; DDF = difficulty describing feelings; DIF = difficulty identifying feelings; EOT = externally-oriented thinking; REAP = cognitive reappraisal; SUPP = expressive suppression

*** *p* < .001

** *p* < .01

* *p* < .05.

In the forensic sample, all coefficients were similar in size and direction, except for suppression and reappraisal, which only yielded non-significant associations with all other variables (suppression: ranging from *r*_forensic_ = –.17 to *r*_forensic_ = .21; *p*s = n.s.; reappraisal: ranging from *r*_forensic_ = –.21 to *r*_forensic_ = .13; *p*s = n.s.).

**Fig 1:**
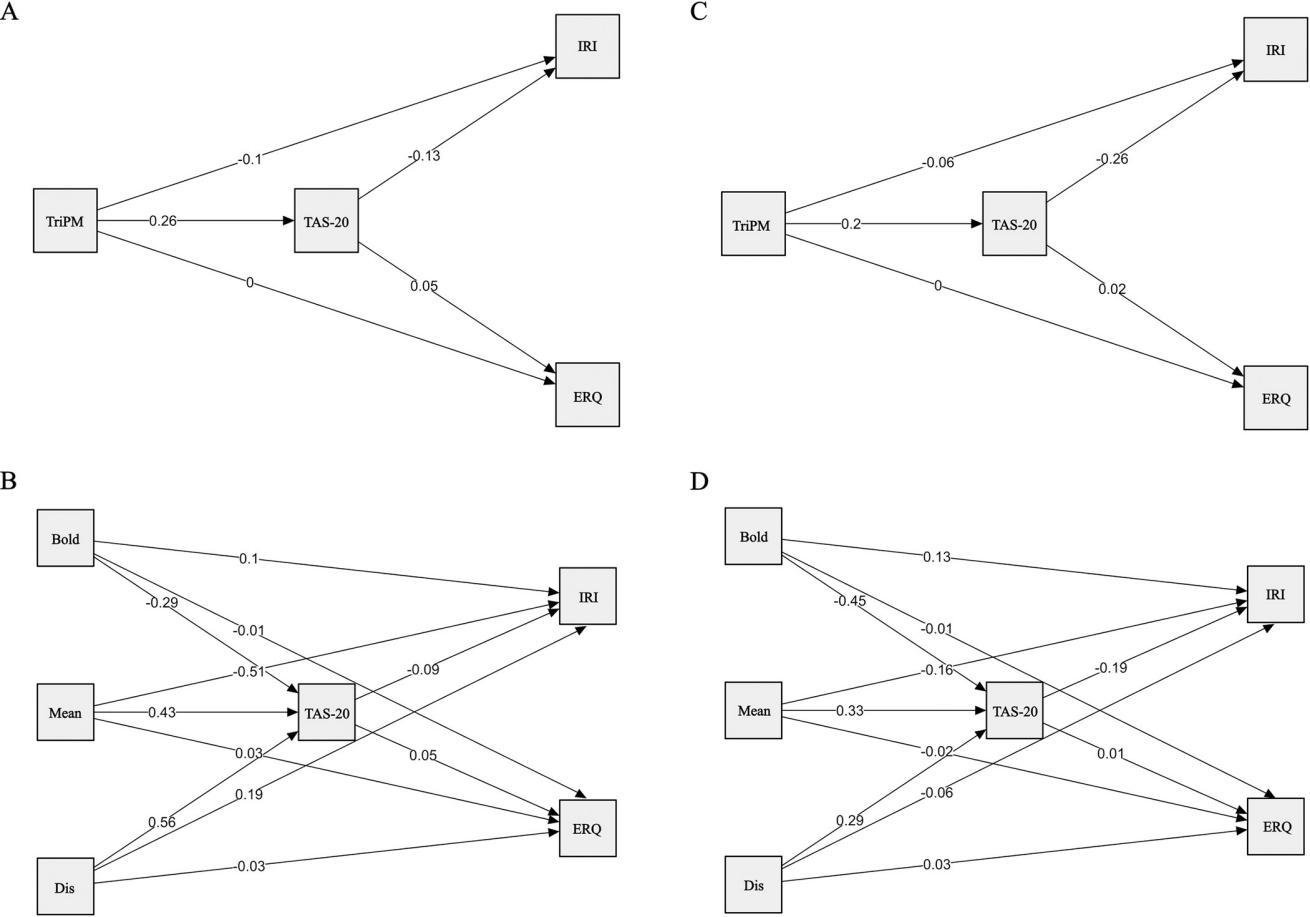
Path models with path coefficients. Model A and B show the results for the community sample (*N* = 315). Model C and D show the results for the forensic sample (*N* = 50). TriPM = Triarchic Psychopathy Measure; TAS = Toronto Alexithymia Scale; IRI = Interpersonal Reactivity Scale; ERQ = Emotion Regulation Questionnaire; Mean = Meanness; Bold = Boldness; Dis = Disinhibition.

The results of the mediation analyses are presented in Tables 5 and 6 (all path coefficients are shown in Fig 1). In the community sample, alexithymia significantly mediated the relationship between psychopathy and empathy (*ab*_*cs*_ = –.073, *p* < .001), and between psychopathy and suppression (*ab*_*cs*_ = .154, *p* < .001). While the former effect was negative and the latter positive, both indirect effects can be considered small (Table 5). In the forensic sample, the direction and magnitude of the two indirect effects were the same, albeit only the mediation from psychopathy to empathy via alexithymia reached statistical significance (*ab*_*cs*_ = –.156, *p* < .05; Table 5). When the three psychopathy factors were included separately in the model, the results changed slightly. In the community sample, all indirect effects remained statistically significant and small, but the mediation from boldness to empathy and suppression through alexithymia yielded effects in the opposite direction (i.e., boldness -> alexithymia -> empathy: *B =* .03, *ab*_*cs*_ = .030, *p* < .05; boldness -> alexithymia -> suppression: *B* = –.02, *ab*_*cs*_ = –.098, *p* < .001; Table 6). Although none of the indirect effects reached statistical significance in the forensic sample, the directions of the effects were consistent with those of the community sample. Due to the complexity of the model along with the limited number of forensic individuals included in the study, these results are reported in the supplementary material (S1 Table). In addition, all mediation analyses were also conducted with reappraisal as an outcome instead of suppression. Since the results were identical to those of suppression but with opposite effects, they are reported for both samples in the supplement (S2–S4 Tables).

**Table 5:** Results of mediation analyses for psychopathy, alexithymia, empathy, and suppression in the forensic and community sample.

Independent Variable (IV)	Mediating Variable (M)	Dependent Variables (DV)	Sample	Effect of IV on M (a)	Effect of M on DV (b)	Direct Effect (c‘)	Total Effect (c)	Indirect effect (a)(b) [95% CI]	Effect size *ab*_*cs*_
Psychopathy	Alexithymia	Empathy	Com	.258***	–.133***	–.104***	–.138***	–.034 [–.061;–.016]***	–.073***
			For	.198**	–.256**	–.062	–.112**	–.051 [–.106;–.016]*	–.156*
		Suppression	Com	.258***	.053***	–.002	.012*	.014 [.008; .020]***	.154***
			For	.198**	.021	.000	.005	.004 [–.001; .014]	.070

Com = Community (*N* = 315); For = Forensic (*N* = 50). Bias-corrected percentile bootstrap confidence intervals (*N* = 5000). Maximum likelihood estimator.

*p < .05

**p < .01

***p < .001.

**Table 6:** Results of mediation analyses for psychopathy factors, alexithymia, empathy, and suppression in the community sample.

Independent Variable (IV)	Mediating Variable (M)	Dependent Variables (DV)	Effect of IV on M (a)	Effect of M on DV (b)	Direct Effect (c‘)	Total Effect (c)	Indirect effect (a)(b) [95% CI]	Effect size *ab*_*cs*_
Meanness	Alexithymia	Empathy	.435***	–.088**	–.515***	–.553***	–.038 [–.089;–.009]*	–.041*
		Suppression	.435***	.054***	.027**	.051***	.023 [.010; .041]***	.131***
Boldness	Alexithymia	Empathy	–.287***	–.088**	.101*	.126**	.025 [.005; .061]*	.030*
		Suppression	–.287***	.054***	–.009	–.025**	–.015 [–.029;–.005]***	–.098***
Disinhibition	Alexithymia	Empathy	.563***	–.088**	.186***	.137*	–.050 [–.104;–.014]*	–.052*
		Suppression	.563***	.054***	–.032**	–.002	.030 [.016; .048]***	.167***

Results are presented for the community sample (*N* = 315). Bias-corrected percentile bootstrap confidence intervals (*N* = 5000). Maximum likelihood estimator. For results on forensic sample, see S1 Table.

*p < .05

**p < .01

***p < .001.

## Discussion

The aim of the present study was to examine the concurrent interrelationship between emotional deficits in psychopathy, with a specific focus on the mediating influence of alexithymia. To this end, we collected data in both a community and a forensic sample. Forensic inpatients reported more psychopathic traits on all three dimensions, irrespective of age, gender, and education. The effect sizes ranged from moderate to large, supporting previous findings demonstrating higher levels of psychopathy in delinquent individuals (for a meta-analysis, see [90]). Surprisingly though, no such differences were found in terms of reported emotional impairments. This is in stark contrast to previous research showing more empathy deficits, alexithymic symptoms, and emotion dysregulation in forensic inpatients compared to healthy individuals [53,55,91,92]. There are three possible explanations for the absence of these differences in our study. First, forensic inpatients may have improved their emotional skills over the course of treatment in the forensic hospital or, on the contrary, learned to simulate them. Second, forensic inpatients may be less aware of their own deficits and believe they are better at describing or regulating their emotions than is actually the case [93]. Third, internal consistencies within the forensic sample were rather poor for some variables, suggesting either issues with comprehending items or potential medication side effects (e.g., sedation), which in turn may have biased responses and thus reduced differences between the two samples.

Our results pertaining to the bivariate associations across all variables are largely consistent with previous studies. In particular, psychopathy was negatively related to empathy and positively related to alexithymia in both samples, as found in a previous meta-analysis [2]. However, the negative and positive relationships between psychopathy and reappraisal as well as between psychopathy and suppression, respectively, were obtained only in the community sample. This contradicts Garofalo et al. [53], who found a negative association between psychopathy and emotion dysregulation in criminal offenders, and cannot be explained solely by the lower power of the forensic sample compared to the community sample. Instead, as noted earlier, this finding may be the result of treatment effects, the unawareness of own internal processes, or the poor alpha coefficients achieved in the forensic sample. A closer examination of the TriPM subscales and their correlations with all measured emotional constructs revealed considerable variation among the three dimensions of psychopathy. Specifically, boldness showed relationships generally in the opposite direction than meanness and disinhibition, namely a positive association with cognitive empathy and reappraisal, and a negative association with alexithymia and suppression. This reinforces the notion that psychopathy should not be viewed as a unitary construct, but rather as a constellation of distinct dimensions [94,95]. Focusing on psychopathy total scores alone may diminish true effects and lead to conflicting research findings [96].

The results of our mediation analyses suggest that the presence of alexithymia accounts for some of the empathy deficits and emotion dysregulation observed in psychopathy. Put simply, psychopathic individuals’ limited understanding of their own feelings contributes to their lack of empathy and tendency to suppress emotions. This aligns well with contemporary theories proposing that the absence of alexithymia is a critical component of healthy empathy and ER skills [11,12,71]. Support also comes from a study by Jonason and Krause [60] who tested a Structural Equation Model that included alexithymia, empathy, and the Dark Triad (i.e., Psychopathy, Narcissism, and Machiavellianism; [97]). Their model revealed an indirect relationship between low empathy and Dark Triad scores mediated through alexithymia. To the best of our knowledge, no study to date has investigated the mediating effect of alexithymia on the association between psychopathy and emotion dysregulation.

Our mediation analyses that considered psychopathy factors (i.e., boldness, disinhibition, meanness) rather than a psychopathy total score yielded the same results for all factors except boldness. More specifically, the indirect effects were inverted, suggesting that boldness is associated with fewer alexithymic symptoms, which in turn attenuates emotion dysregulation and increases empathy. This adds to the view that boldness may be an adaptive feature of psychopathy [95,98–101]. Prior research has already linked boldness to higher heart rate variability [102], lower levels of neuroticism [103], and various other positive outcomes (for a meta-analytical review, see [104]). Our results add to this literature by highlighting the role of alexithymia (or the lack thereof) in shaping the adaptive qualities of boldness and its positive impact on emotional functioning.

It is important to acknowledge that the indirect effects reported in this study are relatively small, and while the direction of these effects were the same in the two samples, the mediating influence of alexithymia on psychopathy and suppression/reappraisal, as well as between all three psychopathy factors and empathy or suppression/reappraisal, did not reach statistical significance in the forensic sample. However, this should by no means be taken as evidence for the absence of a mediating effect of alexithymia. Rather, it is likely due to the limited power of the forensic sample. We therefore encourage future studies to replicate our analyses in larger forensic populations before drawing final conclusions.

## Limitations

Several limitations should be noted when interpreting our results. First and foremost, the poor internal consistencies achieved for some variables in the forensic sample are a concern as they reduce data quality and attenuate the true effects. There are countless causes for low alphas, but the fact that not all variables showed poor internal consistencies, suggests that it was not due to a general lack of motivation on the part of the forensic inpatients. Instead, there may have been an issue with fully comprehending all items. The ERQ in particular has been criticized for its complex wording [105,106], which is supported by our own observations of many inpatients asking for clarification of the meaning of some items.

Second, no data on mental disorders was collected from forensic inpatients. While all individuals included in the forensic sample had a clinical diagnosis of at least one mental disorder, we were unable to control for confounding effects of specific disorders and medications on our results. We therefore suggest that future research explores possible moderating influences of different mental disorders and medications on the associations reported in the present study.

Third, we did not assess treatment intensity and duration within the forensic sample. The primary goal of a forensic hospital is to reduce a patient’s likelihood to recidivate. Treating emotional deficits is an important part of it [107–110]. Thus, it is entirely possible that those who received more treatment were less likely to report emotional deficits, which may have ultimately reduced the observed correlations between psychopathy and emotional deficits in this sample. Conversely, forensic patients may also lack insight into their emotional deficits or attempt to portray themselves in a better light through socially desirable responses. Future studies could therefore improve assessments by including interviews that are less vulnerable to these limitations, such as the Level of Emotional Awareness Scale (LEAS; [111,112]).

Fourth, although we made an effort to include a diverse sample of individuals from the community, our sample is clearly biased toward well-educated and young individuals. This makes it difficult to generalize the findings. Nonetheless, our statistically significant results within this sample suggest that psychopathy can be studied in non-offender samples as long as a tool is used that does not overemphasize criminal behavior.

Finally, we used an online survey in the community sample and a paper-pencil version of our questionnaires in the forensic sample. This disparity in data collection might have affected the results. However, given the comparable correlation coefficients and mediation effects in the two samples, we assume that the influence, if present at all, was weak.

## Implications for future research and clinical practice

The findings of the present study have implications for both future research and clinical practice. Specifically, the low internal consistencies and somewhat unexpected associations observed in the forensic sample suggest that self-report instruments may not always be the best choice to assess emotional deficits in institutionalized individuals. Previous studies have already raised concerns about using self-report questionnaires in forensic populations due to higher levels of social desirability, or difficulties understanding items [106,113]. Future studies may therefore seek to improve by employing more appropriate instruments that are tailored to the respondents’ cognitive abilities and include measures to detect response biases.

It is crucial for future studies to also include state measures of emotional abilities and corresponding psychophysiological markers. By doing so, researchers can gain a better understanding of the situational aspects of emotional deficits in psychopathy and identify external factors that might influence them (e.g., motivation; [114]). Including such measures can provide a more complete picture of the concurrent interrelationship of emotional deficits in psychopathy than self-report trait measures alone.

With regard to clinical practice, our findings suggest that targeting alexithymic symptoms in the treatment of psychopathic individuals could have a positive carryover effect on other emotional deficits. While previous studies have demonstrated the efficacy of alexithymia training in offender populations [107,109], there is currently a lack of research on this approach specifically for psychopathy. This is surprising considering that a lack of emotional awareness has long been recognized as a fundamental characteristic of psychopathy [115]. Thus, clinical trials that evaluate interventions aimed at improving psychopathic individuals’ perception and understanding of their own emotions are urgently needed.

## Conclusion

This is the first study to examine the mediating influence of alexithymia on the relationship between psychopathy and empathy as well as between psychopathy and emotion dysregulation. Our results strongly suggest that alexithymic symptoms account, at least to some extent, for the lack of empathy and emotion dysregulation observed in individuals with psychopathy. Addressing alexithymia during treatment may therefore be a critical component of effective interventions for psychopathy.

## Supporting information

S1 Table Results of mediation analyses for psychopathy factors, alexithymia, empathy, and suppression in the forensic sample. Results are presented for the forensic sample (*N* = 50). Bias-corrected percentile bootstrap confidence intervals (*N* = 5000). Maximum likelihood estimator. *p < .05; **p < .01; ***p < .001.
(DOCX)

S2 Table Results of mediation analyses for psychopathy, alexithymia, empathy, and reappraisal in the forensic and community sample. Com = Community (*N* = 315); For = Forensic (*N* = 50). Bias-corrected percentile bootstrap confidence intervals (*N* = 5000). Maximum likelihood estimator. *p < .05; **p < .01; ***p < .001.
(DOCX)

S3 Table Results of mediation analyses for psychopathy factors, alexithymia, empathy, and reappraisal in the community sample. Results are presented for the community sample (*N* = 315). Bias-corrected percentile bootstrap confidence intervals (*N* = 5000). Maximum likelihood estimator. *p < .05; **p < .01; ***p < .001.
(DOCX)

S4 Table Results of mediation analyses for psychopathy factors, alexithymia, empathy, and reappraisal in the forensic sample. Results are presented for the forensic sample (*N* = 50). Bias-corrected percentile bootstrap confidence intervals (*N* = 5000). Maximum likelihood estimator. *p < .05; **p < .01; ***p < .001.
(DOCX)
