# Supplementary material for: Understanding empathy deficits and emotion dysregulation in psychopathy: The mediating role of alexithymia
Source: PLoS One. 2024 May 8;19(5):e0301085. doi: 10.1371/journal.pone.0301085 (PMC11078418; doi:10.1371/journal.pone.0301085)
Supplement: S1 Table — Results are presented for the forensic sample (N = 50). Bias-corrected percentile bootstrap confidence intervals (N = 5000). Maximum likelihood estimator. *p < .05; **p < .01; ***p < .001. (DOCX) [file pone.0301085.s001.docx]

**S1 Table. Results of mediation analyses for psychopathy factors, alexithymia, empathy, and suppression in the forensic sample.**

| **Independent Variable (IV)** | **Mediating Variable (M)** | **Dependent Variables (DV)** | **Effect of IV on M (a)** | **Effect of M on DV (b)** | **Direct Effect  (c‘)** | **Total Effect  (c)** | **Indirect effect  (a)(b) [95% CI]** | **Effect size *ab_cs_*** |
| --- | --- | --- | --- | --- | --- | --- | --- | --- |
| Meanness | Alexithymia | Empathy | .332 | –.185^*^ | –.160 | –.221 | –.061 [–.231; .008] | –.009 |
|  |  | Suppression | .332 | .013 | –.019 | –.015 | .004 [–.009; .030] | .003 |
| Boldness | Alexithymia | Empathy | –.449^*^ | –.185^*^ | .128 | .211 | .083 [–.004; .260] | .012 |
|  |  | Suppression | –.449^*^ | .013 | –.008 | –.014 | –.006 [–.029; .014] | –.005 |
| Disinhibition | Alexithymia | Empathy | .289 | –.185^*^ | –.064 | –.118 | –.054 [–.171; .004] | –.008 |
|  |  | Suppression | .289 | .013 | .026 | .030 | .004 [–.007; .024] | .003 |

Results are presented for the forensic sample (*N* = 50). Bias-corrected percentile bootstrap confidence intervals (*N* = 5000). Maximum likelihood estimator.

^*^*p* < .05; ^**^*p* < .01; ^***^*p* < .001.
