# Supplementary material for: Understanding empathy deficits and emotion dysregulation in psychopathy: The mediating role of alexithymia
Source: PLoS One. 2024 May 8;19(5):e0301085. doi: 10.1371/journal.pone.0301085 (PMC11078418; doi:10.1371/journal.pone.0301085)
Supplement: S2 Table — Com = Community (N = 315); For = Forensic (N = 50). Bias-corrected percentile bootstrap confidence intervals (N = 5000). Maximum likelihood estimator. *p < .05; **p < .01; ***p < .001. (DOCX) [file pone.0301085.s002.docx]

**S2 Table. Results of mediation analyses for psychopathy, alexithymia, empathy, and reappraisal in the forensic and community sample***.*

| **Independent Variable (IV)** | **Mediating Variable (M)** | **Dependent Variables (DV)** | **Sample** | **Effect of IV on M (a)** | **Effect of M on DV (b)** | **Direct Effect  (c‘)** | **Total Effect  (c)** | **Indirect effect  (a)(b) [95% CI]** | **Effect size *ab_cs_*** |
| --- | --- | --- | --- | --- | --- | --- | --- | --- | --- |
| Psychopathy | Alexithymia | Empathy | Com | .258^***^ | –.133^***^ | –.104^***^ | –.138^***^ | –.034 [–.060; –.016]^***^ | –.005^***^ |
|  |  |  | For | .198^**^ | –.256^**^ | –.062 | –.112^**^ | –.051 [–.110; –.015]^*^ | –.007^*^ |
|  |  | Reappraisal | Com | .258^***^ | –.029^***^ | –.003 | –.011^*^ | –.007 [–.012; –.004]^***^ | –.007^*^ |
|  |  |  | For | .198^**^ | –.009 | .007 | .005 | –.002 [–.012; .007] | –.001 |

Com = Community (*N* = 315); For = Forensic (*N* = 50). Bias-corrected percentile bootstrap confidence intervals (*N* = 5000). Maximum likelihood estimator.

^*^*p* < .05; ^**^*p* < .01; ^***^*p* < .001.
