# Supplementary material for: Understanding empathy deficits and emotion dysregulation in psychopathy: The mediating role of alexithymia
Source: PLoS One. 2024 May 8;19(5):e0301085. doi: 10.1371/journal.pone.0301085 (PMC11078418; doi:10.1371/journal.pone.0301085)
Supplement: S3 Table — Results are presented for the community sample (N = 315). Bias-corrected percentile bootstrap confidence intervals (N = 5000). Maximum likelihood estimator. *p < .05; **p < .01; ***p < .001. (DOCX) [file pone.0301085.s003.docx]

**S3 Table. Results of mediation analyses for psychopathy factors, alexithymia, empathy, and reappraisal in the community sample.**

| **Independent Variable (IV)** | **Mediating Variable (M)** | **Dependent Variables (DV)** | **Effect of IV on M (a)** | **Effect of M on DV (b)** | **Direct Effect  (c‘)** | **Total Effect  (c)** | **Indirect effect  (a)(b) [95% CI]** | **Effect size *ab_cs_*** |
| --- | --- | --- | --- | --- | --- | --- | --- | --- |
| Meanness | Alexithymia | Empathy | .435^***^ | –.088^*^ | –.515^***^ | –.553^***^ | –.038 [–.092; –.009]^*^ | –.006^*^ |
|  |  | Reappraisal | .435^***^ | –.023^***^ | –.033^***^ | –.043^***^ | –.010 [–.019; –.004]^**^ | –.009^**^ |
| Boldness | Alexithymia | Empathy | –.287^***^ | –.088^*^ | .101^*^ | .0126^**^ | .025 [.005; .062]^*^ | .004^*^ |
|  |  | Reappraisal | –.287^***^ | –.023^***^ | .016^*^ | .023^**^ | .007 [.002; .013]^**^ | .006^**^ |
| Disinhibition | Alexithymia | Empathy | .563^***^ | –.088^*^ | .0186^***^ | .0137^*^ | –.050 [–.106; –.015]^*^ | –.007^*^ |
|  |  | Reappraisal | .563^***^ | –.023^***^ | .012 | –.001 | –.013 [–.025; –.006]^***^ | –.012^***^ |

Results are presented for the community sample (*N* = 315). Bias-corrected percentile bootstrap confidence intervals (*N* = 5000). Maximum likelihood estimator.

^*^*p* < .05; ^**^*p* < .01; ^***^*p* < .001.
